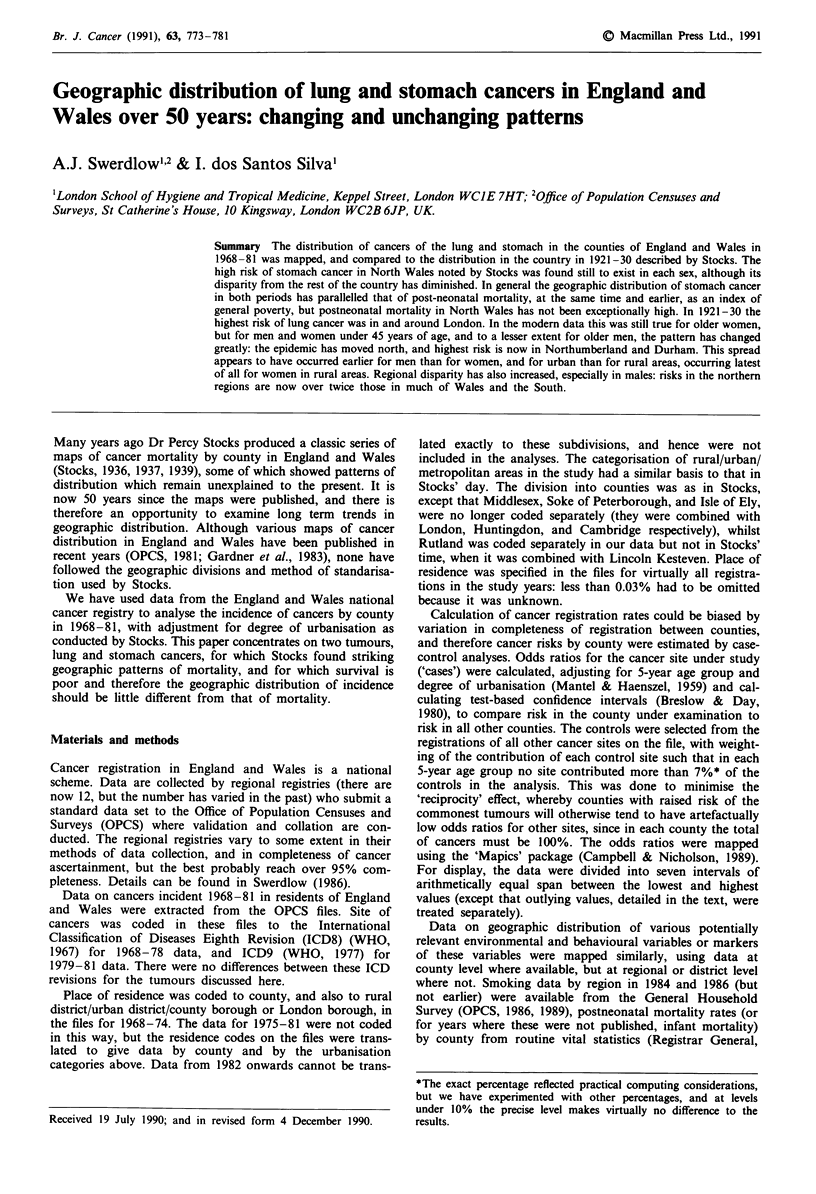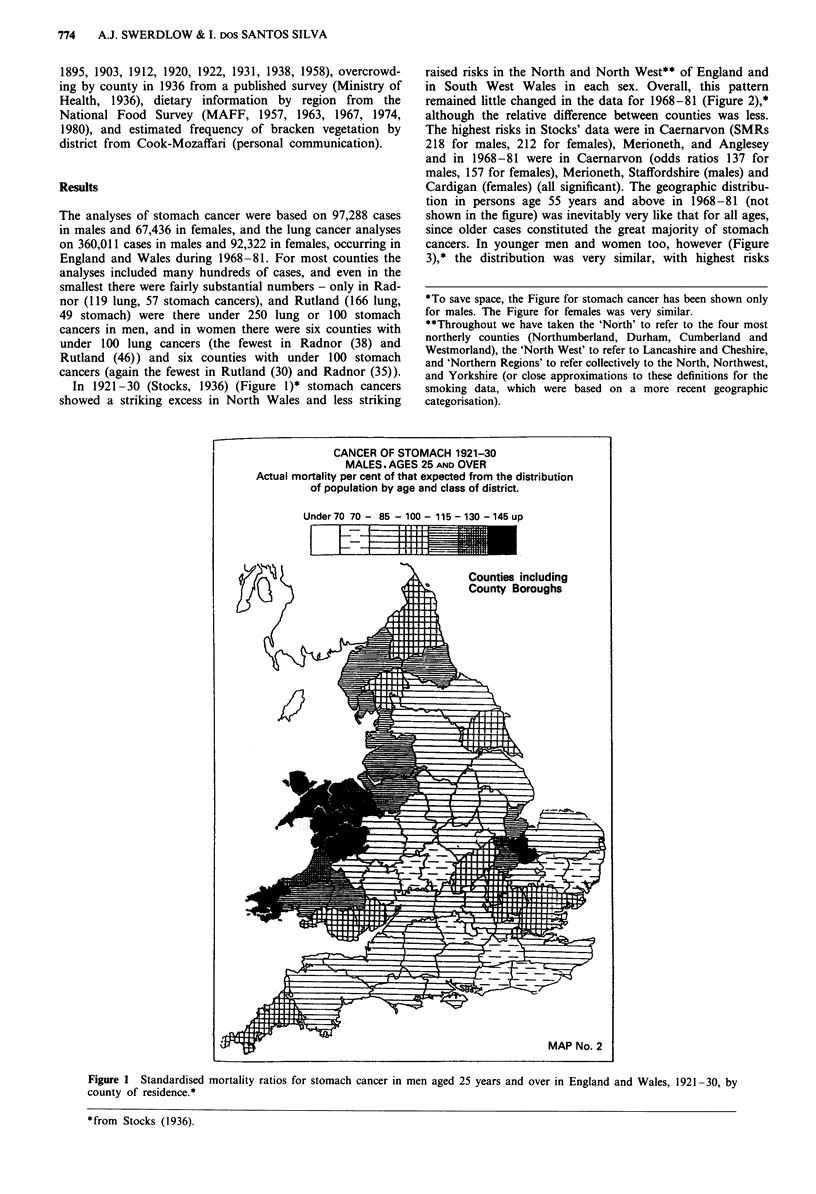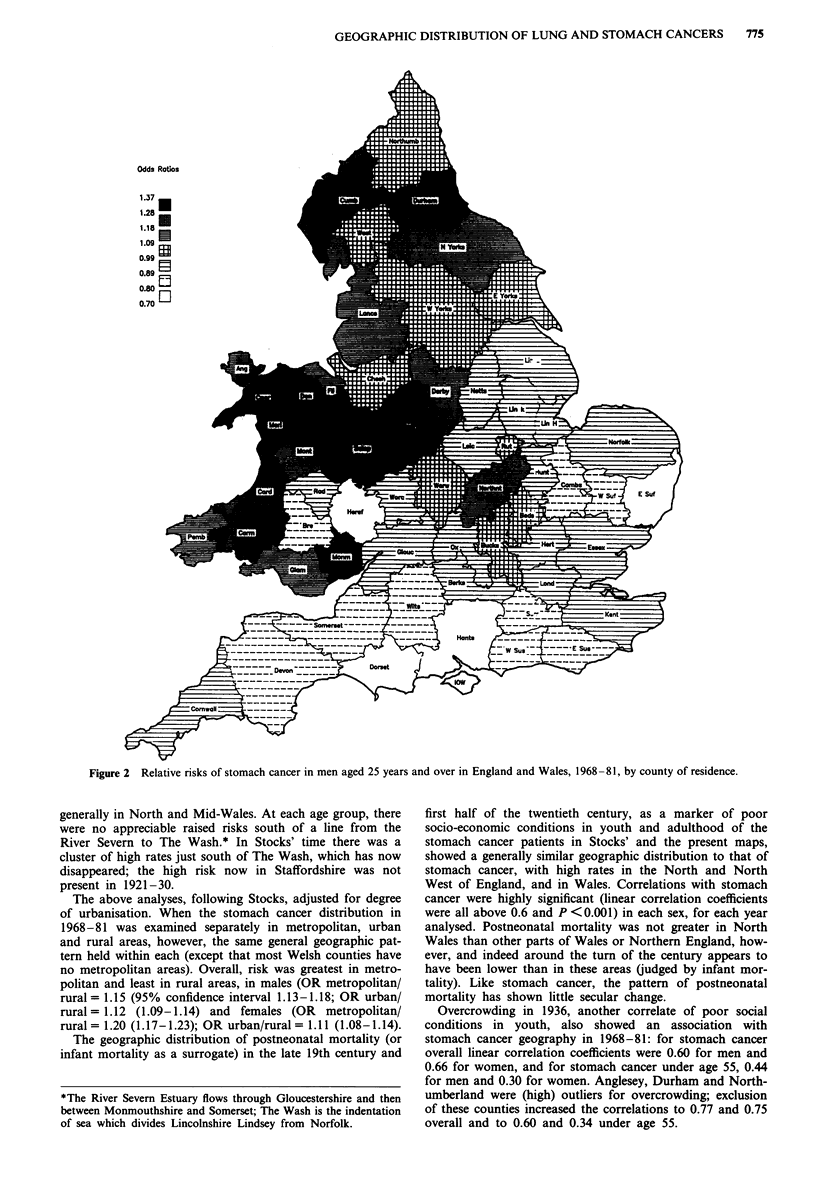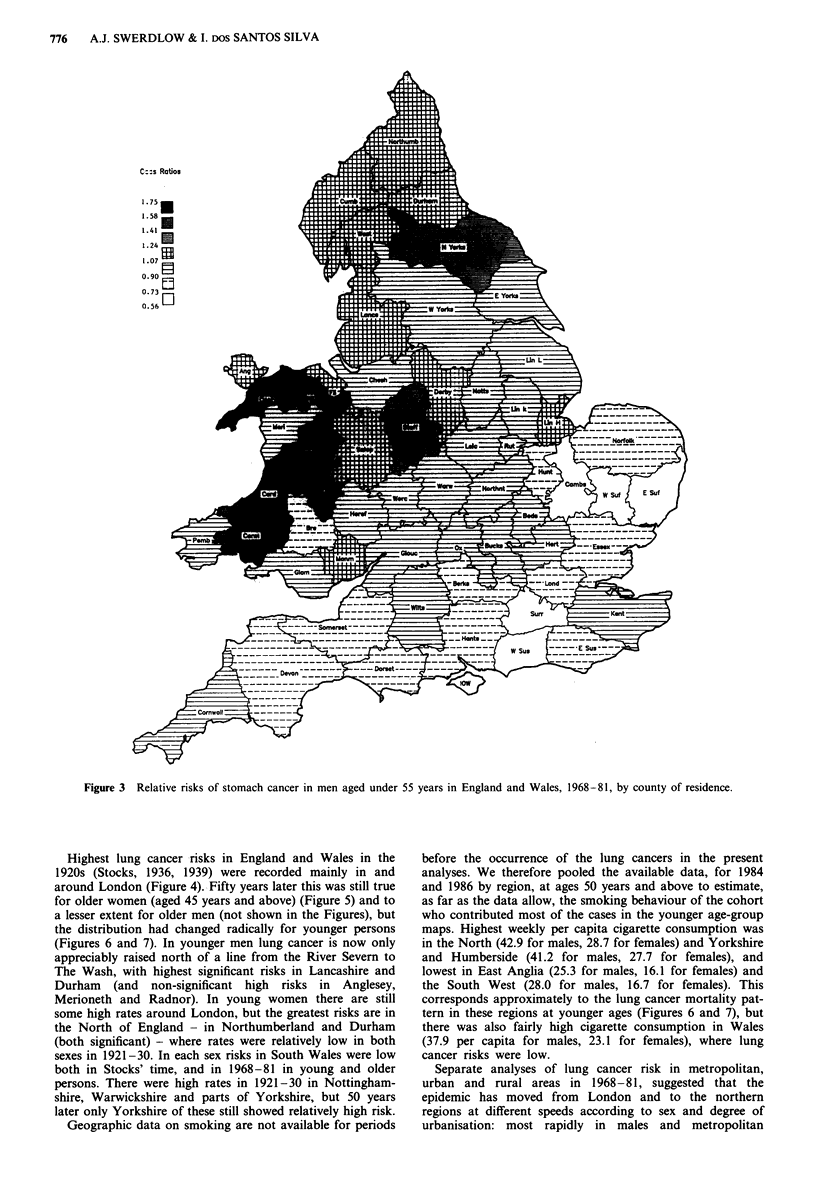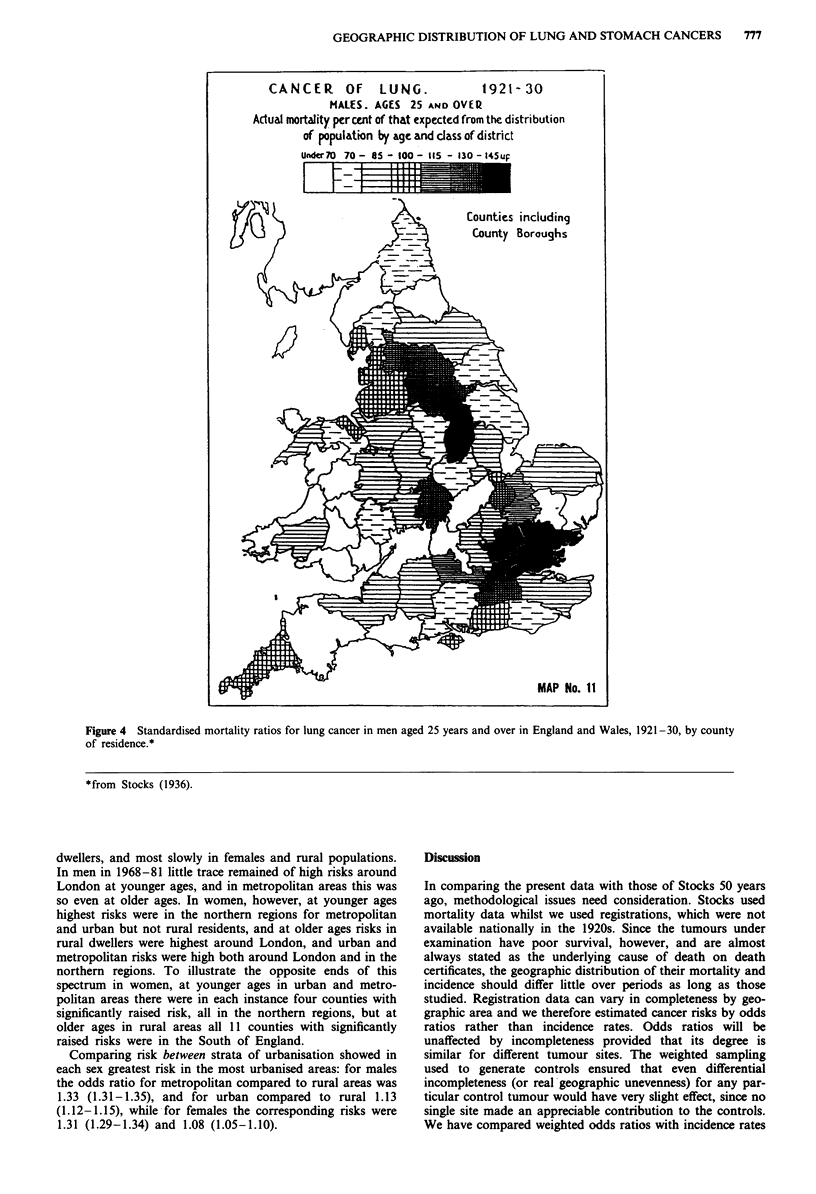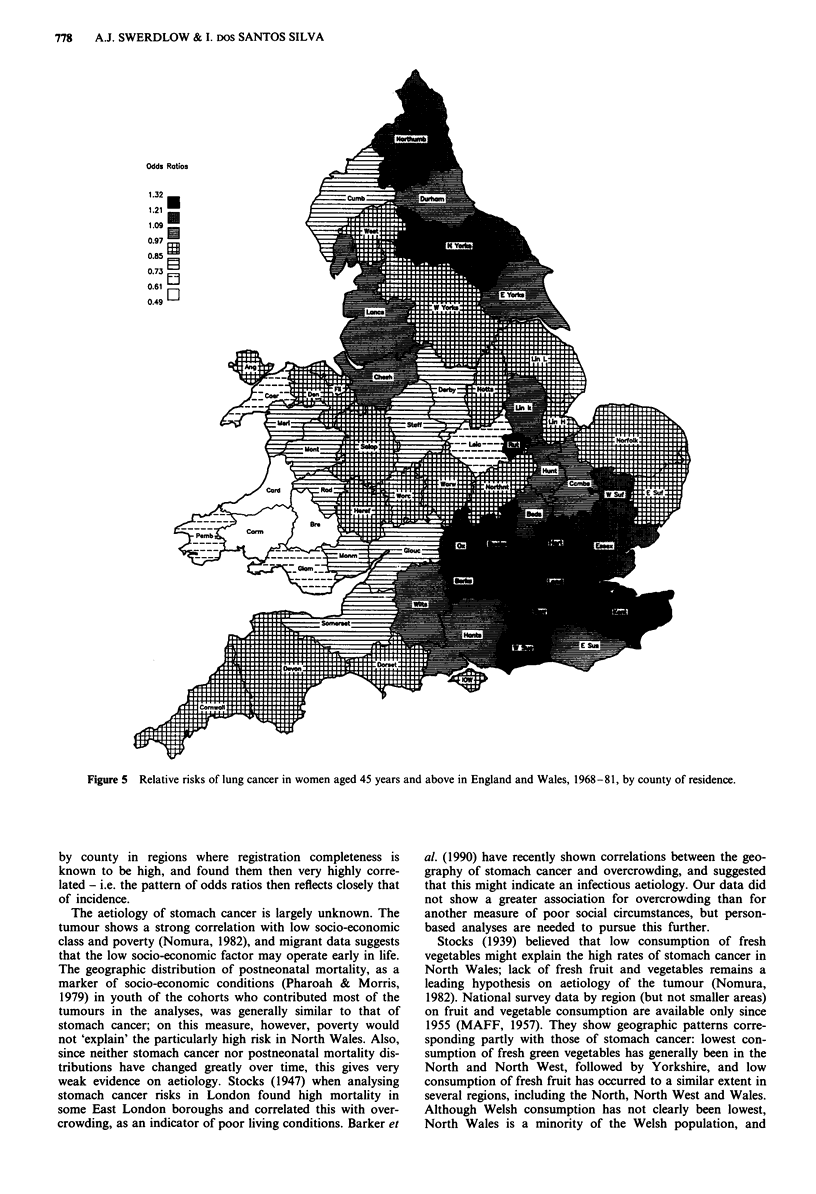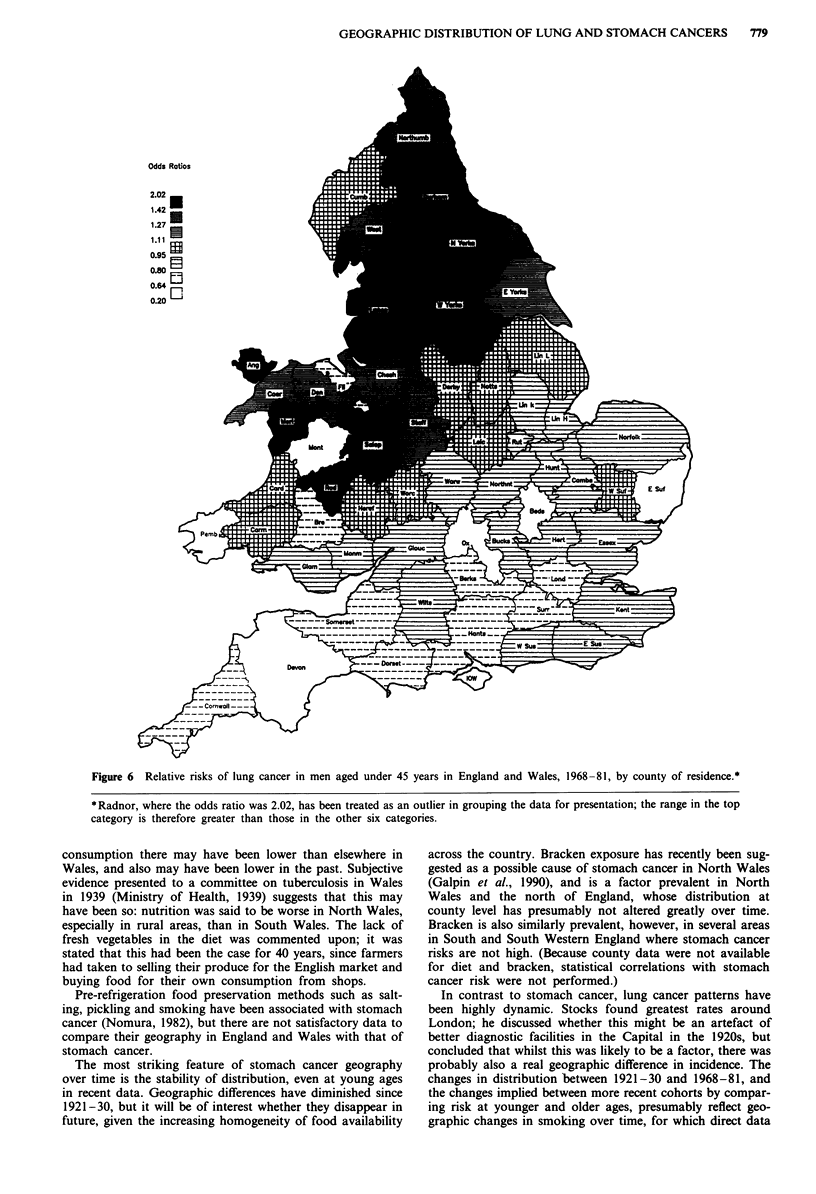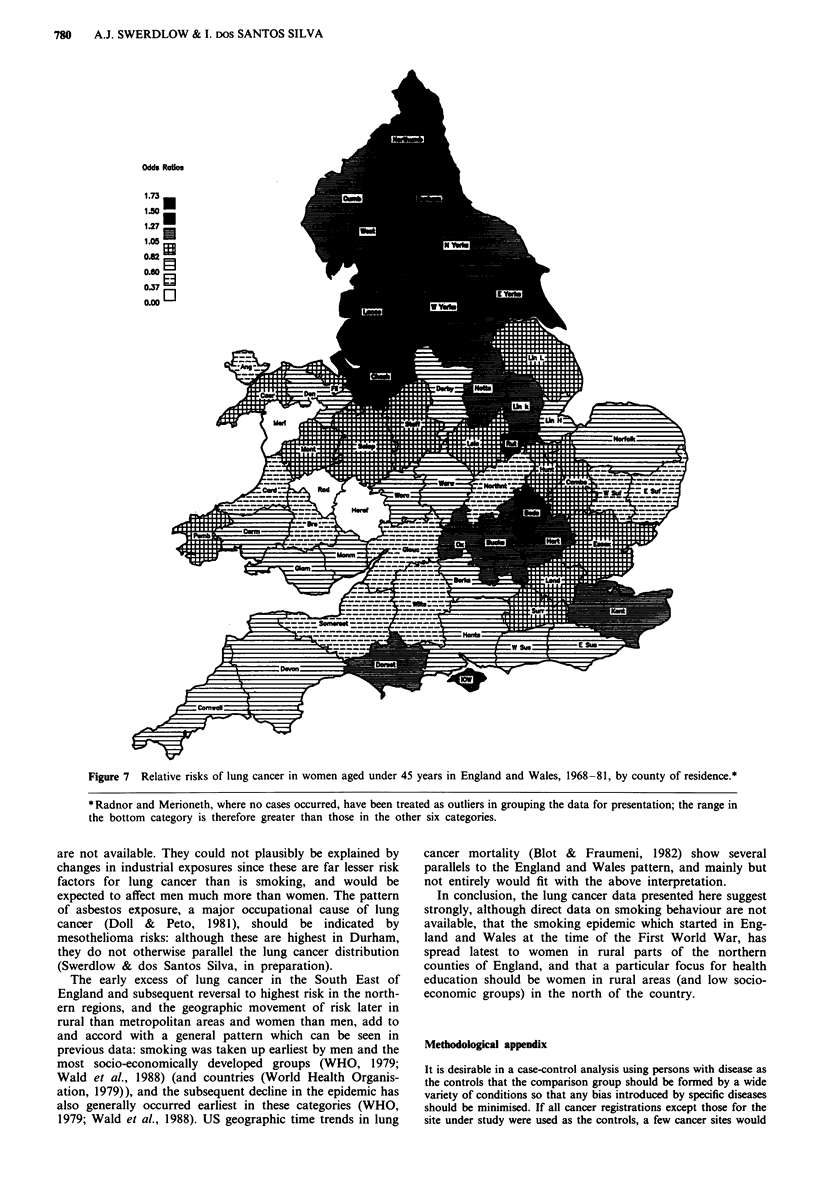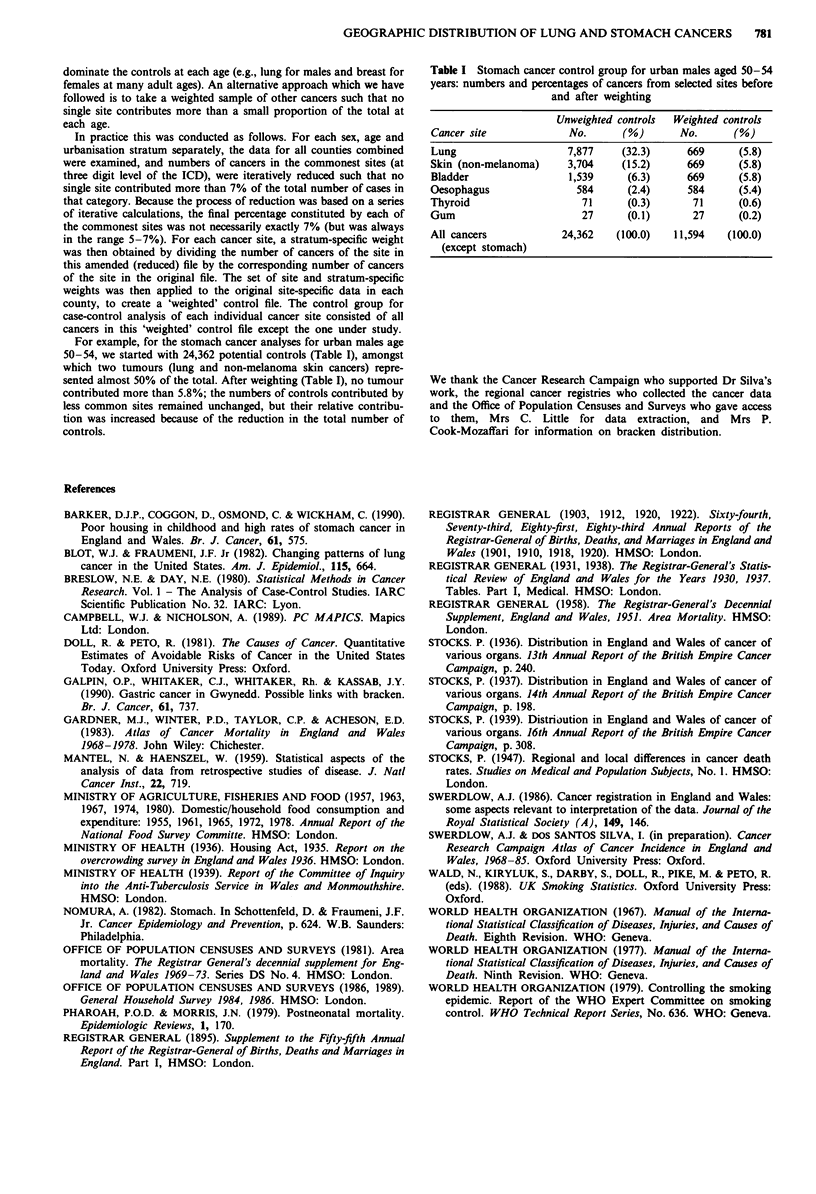# Geographic distribution of lung and stomach cancers in England and Wales over 50 years: changing and unchanging patterns.

**DOI:** 10.1038/bjc.1991.173

**Published:** 1991-05

**Authors:** A. J. Swerdlow, I. dos Santos Silva

**Affiliations:** London School of Hygiene and Tropical Medicine, UK.

## Abstract

The distribution of cancers of the lung and stomach in the counties of England and Wales in 1968-81 was mapped, and compared to the distribution in the country in 1921-30 described by Stocks. The high risk of stomach cancer in North Wales noted by Stocks was found still to exist in each sex, although its disparity from the rest of the country has diminished. In general the geographic distribution of stomach cancer in both periods has paralleled that of post-neonatal mortality, at the same time and earlier, as an index of general poverty, but postneonatal mortality in North Wales has not been exceptionally high. In 1921-30 the highest risk of lung cancer was in and around London. In the modern data this was still true for older women, but for men and women under 45 years of age, and to a lesser extent for older men, the pattern has changed greatly; the epidemic has moved north, and highest risk is now in Northumberland and Durham. This spread appears to have occurred earlier for men than for women, and for urban than for rural areas, occurring latest of all for women in rural areas. Regional disparity has also increased, especially in males: risks in the northern regions are now over twice those in much of Wales and the South.


Br. J. Cancer (1991), 63, 773-781 © Macmillan Press Ltd., 1991

### Geographic distribution of lung and stomach cancers in England and Wales over 50 years: changing and unchanging patterns

A.J. Swerdlow[1,2] & I. dos Santos Silva[1]

[1]*London School of Hygiene and Tropical Medicine, Keppel Street, London WC1E 7HT;* [2]*Office of Population Censuses and Surveys, St Catherine's House, 10 Kingsway, London WC2B 6JP, UK.*

**Summary** The distribution of cancers of the lung and stomach in the counties of England and Wales in 1968-81 was mapped, and compared to the distribution in the country in 1921-30 described by Stocks. The high risk of stomach cancer in North Wales noted by Stocks was found still to exist in each sex, although its disparity from the rest of the country has diminished. In general the geographic distribution of stomach cancer in both periods has parallelled that of post-neonatal mortality, at the same time and earlier, as an index of general poverty, but postneonatal mortality in North Wales has not been exceptionally high. In 1921-30 the highest risk of lung cancer was in and around London. In the modern data this was still true for older women, but for men and women under 45 years of age, and to a lesser extent for older men, the pattern has changed greatly: the epidemic has moved north, and highest risk is now in Northumberland and Durham. This spread appears to have occurred earlier for men than for women, and for urban than for rural areas, occurring latest of all for women in rural areas. Regional disparity has also increased, especially in males: risks in the northern regions are now over twice those in much of Wales and the South.

Many years ago Dr Percy Stocks produced a classic series of maps of cancer mortality by county in England and Wales (Stocks, 1936, 1937, 1939), some of which showed patterns of distribution which remain unexplained to the present. It is now 50 years since the maps were published, and there is therefore an opportunity to examine long term trends in geographic distribution. Although various maps of cancer distribution in England and Wales have been published in recent years (OPCS, 1981; Gardner *et al.*, 1983), none have followed the geographic divisions and method of standarisation used by Stocks.

We have used data from the England and Wales national cancer registry to analyse the incidence of cancers by county in 1968-81, with adjustment for degree of urbanisation as conducted by Stocks. This paper concentrates on two tumours, lung and stomach cancers, for which Stocks found striking geographic patterns of mortality, and for which survival is poor and therefore the geographic distribution of incidence should be little different from that of mortality.

### Materials and methods

Cancer registration in England and Wales is a national scheme. Data are collected by regional registries (there are now 12, but the number has varied in the past) who submit a standard data set to the Office of Population Censuses and Surveys (OPCS) where validation and collation are conducted. The regional registries vary to some extent in their methods of data collection, and in completeness of cancer ascertainment, but the best probably reach over 95% completeness. Details can be found in Swerdlow (1986).

Data on cancers incident 1968-81 in residents of England and Wales were extracted from the OPCS files. Site of cancers was coded in these files to the International Classification of Diseases Eighth Revision (ICD8) (WHO, 1967) for 1968-78 data, and ICD9 (WHO, 1977) for 1979-81 data. There were no differences between these ICD revisions for the tumours discussed here.

Place of residence was coded to county, and also to rural district/urban district/county borough or London borough, in the files for 1968-74. The data for 1975-81 were not coded in this way, but the residence codes on the files were translated to give data by county and by the urbanisation categories above. Data from 1982 onwards cannot be translated exactly to these subdivisions, and hence were not included in the analyses. The categorisation of rural/urban/metropolitan areas in the study had a similar basis to that in Stocks' day. The division into counties was as in Stocks, except that Middlesex, Soke of Peterborough, and Isle of Ely, were no longer coded separately (they were combined with London, Huntingdon, and Cambridge respectively), whilst Rutland was coded separately in our data but not in Stocks' time, when it was combined with Lincoln Kesteven. Place of residence was specified in the files for virtually all registrations in the study years: less than 0.03% had to be omitted because it was unknown.

Calculation of cancer registration rates could be biased by variation in completeness of registration between counties, and therefore cancer risks by county were estimated by case-control analyses. Odds ratios for the cancer site under study ('cases') were calculated, adjusting for 5-year age group and degree of urbanisation (Mantel & Haenszel, 1959) and calculating test-based confidence intervals (Breslow & Day, 1980), to compare risk in the county under examination to risk in all other counties. The controls were selected from the registrations of all other cancer sites on the file, with weighting of the contribution of each control site such that in each 5-year age group no site contributed more than 7%* of the controls in the analysis. This was done to minimise the 'reciprocity' effect, whereby counties with raised risk of the commonest tumours will otherwise tend to have artefactually low odds ratios for other sites, since in each county the total of cancers must be 100%. The odds ratios were mapped using the 'Mapics' package (Campbell & Nicholson, 1989). For display, the data were divided into seven intervals of arithmetically equal span between the lowest and highest values (except that outlying values, detailed in the text, were treated separately).

Data on geographic distribution of various potentially relevant environmental and behavioural variables or markers of these variables were mapped similarly, using data at county level where available, but at regional or district level where not. Smoking data by region in 1984 and 1986 (but not earlier) were available from the General Household Survey (OPCS, 1986, 1989), postneonatal mortality rates (or for years where these were not published, infant mortality) by county from routine vital statistics (Registrar General,

Received 19 July 1990; and in revised form 4 December 1990.

*The exact percentage reflected practical computing considerations, but we have experimented with other percentages, and at levels under 10% the precise level makes virtually no difference to the results.

1895, 1903, 1912, 1920, 1922, 1931, 1938, 1958), overcrowding by county in 1936 from a published survey (Ministry of Health, 1936), dietary information by region from the National Food Survey (MAFF, 1957, 1963, 1967, 1974, 1980), and estimated frequency of bracken vegetation by district from Cook-Mozaffari (personal communication).

### Results

The analyses of stomach cancer were based on 97,288 cases in males and 67,436 in females, and the lung cancer analyses on 360,011 cases in males and 92,322 in females, occurring in England and Wales during 1968–81. For most counties the analyses included many hundreds of cases, and even in the smallest there were fairly substantial numbers – only in Radnor (119 lung, 57 stomach cancers), and Rutland (166 lung, 49 stomach) were there under 250 lung or 100 stomach cancers in men, and in women there were six counties with under 100 lung cancers (the fewest in Radnor (38) and Rutland (46)) and six counties with under 100 stomach cancers (again the fewest in Rutland (30) and Radnor (35)).

In 1921–30 (Stocks, 1936) (Figure 1)* stomach cancers showed a striking excess in North Wales and less striking raised risks in the North and North West** of England and in South West Wales in each sex. Overall, this pattern remained little changed in the data for 1968–81 (Figure 2),* although the relative difference between counties was less. The highest risks in Stocks' data were in Caernarvon (SMRs 218 for males, 212 for females), Merioneth, and Anglesey and in 1968–81 were in Caernarvon (odds ratios 137 for males, 157 for females), Merioneth, Staffordshire (males) and Cardigan (females) (all significant). The geographic distribution in persons age 55 years and above in 1968–81 (not shown in the figure) was inevitably very like that for all ages, since older cases constituted the great majority of stomach cancers. In younger men and women too, however (Figure 3),* the distribution was very similar, with highest risks

*To save space, the Figure for stomach cancer has been shown only for males. The Figure for females was very similar.

**Throughout we have taken the 'North' to refer to the four most northerly counties (Northumberland, Durham, Cumberland and Westmorland), the 'North West' to refer to Lancashire and Cheshire, and 'Northern Regions' to refer collectively to the North, Northwest, and Yorkshire (or close approximations to these definitions for the smoking data, which were based on a more recent geographic categorisation).

**Figure 1** Standardised mortality ratios for stomach cancer in men aged 25 years and over in England and Wales, 1921–30, by county of residence.*

*from Stocks (1936).

**Figure 2** Relative risks of stomach cancer in men aged 25 years and over in England and Wales, 1968–81, by county of residence.

generally in North and Mid-Wales. At each age group, there were no appreciable raised risks south of a line from the River Severn to The Wash.* In Stocks' time there was a cluster of high rates just south of The Wash, which has now disappeared; the high risk now in Staffordshire was not present in 1921–30.

The above analyses, following Stocks, adjusted for degree of urbanisation. When the stomach cancer distribution in 1968–81 was examined separately in metropolitan, urban and rural areas, however, the same general geographic pattern held within each (except that most Welsh counties have no metropolitan areas). Overall, risk was greatest in metropolitan and least in rural areas, in males (OR metropolitan/rural = 1.15 (95% confidence interval 1.13–1.18; OR urban/rural = 1.12 (1.09–1.14) and females (OR metropolitan/rural = 1.20 (1.17–1.23); OR urban/rural = 1.11 (1.08–1.14).

The geographic distribution of postneonatal mortality (or infant mortality as a surrogate) in the late 19th century and first half of the twentieth century, as a marker of poor socio-economic conditions in youth and adulthood of the stomach cancer patients in Stocks' and the present maps, showed a generally similar geographic distribution to that of stomach cancer, with high rates in the North and North West of England, and in Wales. Correlations with stomach cancer were highly significant (linear correlation coefficients were all above 0.6 and $P < 0.001$) in each sex, for each year analysed. Postneonatal mortality was not greater in North Wales than other parts of Wales or Northern England, however, and indeed around the turn of the century appears to have been lower than in these areas (judged by infant mortality). Like stomach cancer, the pattern of postneonatal mortality has shown little secular change.

Overcrowding in 1936, another correlate of poor social conditions in youth, also showed an association with stomach cancer geography in 1968–81: for stomach cancer overall linear correlation coefficients were 0.60 for men and 0.66 for women, and for stomach cancer under age 55, 0.44 for men and 0.30 for women. Anglesey, Durham and Northumberland were (high) outliers for overcrowding; exclusion of these counties increased the correlations to 0.77 and 0.75 overall and to 0.60 and 0.34 under age 55.

*The River Severn Estuary flows through Gloucestershire and then between Monmouthshire and Somerset; The Wash is the indentation of sea which divides Lincolnshire Lindsey from Norfolk.

**Figure 3** Relative risks of stomach cancer in men aged under 55 years in England and Wales, 1968–81, by county of residence.

Highest lung cancer risks in England and Wales in the 1920s (Stocks, 1936, 1939) were recorded mainly in and around London (Figure 4). Fifty years later this was still true for older women (aged 45 years and above) (Figure 5) and to a lesser extent for older men (not shown in the Figures), but the distribution had changed radically for younger persons (Figures 6 and 7). In younger men lung cancer is now only appreciably raised north of a line from the River Severn to The Wash, with highest significant risks in Lancashire and Durham (and non-significant high risks in Anglesey, Merioneth and Radnor). In young women there are still some high rates around London, but the greatest risks are in the North of England – in Northumberland and Durham (both significant) – where rates were relatively low in both sexes in 1921–30. In each sex risks in South Wales were low both in Stocks' time, and in 1968–81 in young and older persons. There were high rates in 1921–30 in Nottinghamshire, Warwickshire and parts of Yorkshire, but 50 years later only Yorkshire of these still showed relatively high risk.

Geographic data on smoking are not available for periods before the occurrence of the lung cancers in the present analyses. We therefore pooled the available data, for 1984 and 1986 by region, at ages 50 years and above to estimate, as far as the data allow, the smoking behaviour of the cohort who contributed most of the cases in the younger age-group maps. Highest weekly per capita cigarette consumption was in the North (42.9 for males, 28.7 for females) and Yorkshire and Humberside (41.2 for males, 27.7 for females), and lowest in East Anglia (25.3 for males, 16.1 for females) and the South West (28.0 for males, 16.7 for females). This corresponds approximately to the lung cancer mortality pattern in these regions at younger ages (Figures 6 and 7), but there was also fairly high cigarette consumption in Wales (37.9 per capita for males, 23.1 for females), where lung cancer risks were low.

Separate analyses of lung cancer risk in metropolitan, urban and rural areas in 1968–81, suggested that the epidemic has moved from London and to the northern regions at different speeds according to sex and degree of urbanisation: most rapidly in males and metropolitan

**Figure 4** Standardised mortality ratios for lung cancer in men aged 25 years and over in England and Wales, 1921–30, by county of residence.*

*from Stocks (1936).

dwellers, and most slowly in females and rural populations. In men in 1968–81 little trace remained of high risks around London at younger ages, and in metropolitan areas this was so even at older ages. In women, however, at younger ages highest risks were in the northern regions for metropolitan and urban but not rural residents, and at older ages risks in rural dwellers were highest around London, and urban and metropolitan risks were high both around London and in the northern regions. To illustrate the opposite ends of this spectrum in women, at younger ages in urban and metropolitan areas there were in each instance four counties with significantly raised risk, all in the northern regions, but at older ages in rural areas all 11 counties with significantly raised risks were in the South of England.

Comparing risk *between* strata of urbanisation showed in each sex greatest risk in the most urbanised areas: for males the odds ratio for metropolitan compared to rural areas was 1.33 (1.31–1.35), and for urban compared to rural 1.13 (1.12–1.15), while for females the corresponding risks were 1.31 (1.29–1.34) and 1.08 (1.05–1.10).

### Discussion

In comparing the present data with those of Stocks 50 years ago, methodological issues need consideration. Stocks used mortality data whilst we used registrations, which were not available nationally in the 1920s. Since the tumours under examination have poor survival, however, and are almost always stated as the underlying cause of death on death certificates, the geographic distribution of their mortality and incidence should differ little over periods as long as those studied. Registration data can vary in completeness by geographic area and we therefore estimated cancer risks by odds ratios rather than incidence rates. Odds ratios will be unaffected by incompleteness provided that its degree is similar for different tumour sites. The weighted sampling used to generate controls ensured that even differential incompleteness (or real geographic unevenness) for any particular control tumour would have very slight effect, since no single site made an appreciable contribution to the controls. We have compared weighted odds ratios with incidence rates

**Figure 5** Relative risks of lung cancer in women aged 45 years and above in England and Wales, 1968–81, by county of residence.

by county in regions where registration completeness is known to be high, and found them then very highly correlated – i.e. the pattern of odds ratios then reflects closely that of incidence.

The aetiology of stomach cancer is largely unknown. The tumour shows a strong correlation with low socio-economic class and poverty (Nomura, 1982), and migrant data suggests that the low socio-economic factor may operate early in life. The geographic distribution of postneonatal mortality, as a marker of socio-economic conditions (Pharoah & Morris, 1979) in youth of the cohorts who contributed most of the tumours in the analyses, was generally similar to that of stomach cancer; on this measure, however, poverty would not 'explain' the particularly high risk in North Wales. Also, since neither stomach cancer nor postneonatal mortality distributions have changed greatly over time, this gives very weak evidence on aetiology. Stocks (1947) when analysing stomach cancer risks in London found high mortality in some East London boroughs and correlated this with overcrowding, as an indicator of poor living conditions. Barker *et al.* (1990) have recently shown correlations between the geography of stomach cancer and overcrowding, and suggested that this might indicate an infectious aetiology. Our data did not show a greater association for overcrowding than for another measure of poor social circumstances, but person-based analyses are needed to pursue this further.

Stocks (1939) believed that low consumption of fresh vegetables might explain the high rates of stomach cancer in North Wales; lack of fresh fruit and vegetables remains a leading hypothesis on aetiology of the tumour (Nomura, 1982). National survey data by region (but not smaller areas) on fruit and vegetable consumption are available only since 1955 (MAFF, 1957). They show geographic patterns corresponding partly with those of stomach cancer: lowest consumption of fresh green vegetables has generally been in the North and North West, followed by Yorkshire, and low consumption of fresh fruit has occurred to a similar extent in several regions, including the North, North West and Wales. Although Welsh consumption has not clearly been lowest, North Wales is a minority of the Welsh population, and

**Figure 6** Relative risks of lung cancer in men aged under 45 years in England and Wales, 1968–81, by county of residence.*

*Radnor, where the odds ratio was 2.02, has been treated as an outlier in grouping the data for presentation; the range in the top category is therefore greater than those in the other six categories.

consumption there may have been lower than elsewhere in Wales, and also may have been lower in the past. Subjective evidence presented to a committee on tuberculosis in Wales in 1939 (Ministry of Health, 1939) suggests that this may have been so: nutrition was said to be worse in North Wales, especially in rural areas, than in South Wales. The lack of fresh vegetables in the diet was commented upon; it was stated that this had been the case for 40 years, since farmers had taken to selling their produce for the English market and buying food for their own consumption from shops.

Pre-refrigeration food preservation methods such as salting, pickling and smoking have been associated with stomach cancer (Nomura, 1982), but there are not satisfactory data to compare their geography in England and Wales with that of stomach cancer.

The most striking feature of stomach cancer geography over time is the stability of distribution, even at young ages in recent data. Geographic differences have diminished since 1921–30, but it will be of interest whether they disappear in future, given the increasing homogeneity of food availability across the country. Bracken exposure has recently been suggested as a possible cause of stomach cancer in North Wales (Galpin *et al.*, 1990), and is a factor prevalent in North Wales and the north of England, whose distribution at county level has presumably not altered greatly over time. Bracken is also similarly prevalent, however, in several areas in South and South Western England where stomach cancer risks are not high. (Because county data were not available for diet and bracken, statistical correlations with stomach cancer risk were not performed.)

In contrast to stomach cancer, lung cancer patterns have been highly dynamic. Stocks found greatest rates around London; he discussed whether this might be an artefact of better diagnostic facilities in the Capital in the 1920s, but concluded that whilst this was likely to be a factor, there was probably also a real geographic difference in incidence. The changes in distribution between 1921–30 and 1968–81, and the changes implied between more recent cohorts by comparing risk at younger and older ages, presumably reflect geographic changes in smoking over time, for which direct data

**Figure 7** Relative risks of lung cancer in women aged under 45 years in England and Wales, 1968–81, by county of residence.*

*Radnor and Merioneth, where no cases occurred, have been treated as outliers in grouping the data for presentation; the range in the bottom category is therefore greater than those in the other six categories.

are not available. They could not plausibly be explained by changes in industrial exposures since these are far lesser risk factors for lung cancer than is smoking, and would be expected to affect men much more than women. The pattern of asbestos exposure, a major occupational cause of lung cancer (Doll & Peto, 1981), should be indicated by mesothelioma risks: although these are highest in Durham, they do not otherwise parallel the lung cancer distribution (Swerdlow & dos Santos Silva, in preparation).

The early excess of lung cancer in the South East of England and subsequent reversal to highest risk in the northern regions, and the geographic movement of risk later in rural than metropolitan areas and women than men, add to and accord with a general pattern which can be seen in previous data: smoking was taken up earliest by men and the most socio-economically developed groups (WHO, 1979; Wald *et al.*, 1988) (and countries (World Health Organisation, 1979)), and the subsequent decline in the epidemic has also generally occurred earliest in these categories (WHO, 1979; Wald *et al.*, 1988). US geographic time trends in lung cancer mortality (Blot & Fraumeni, 1982) show several parallels to the England and Wales pattern, and mainly but not entirely would fit with the above interpretation.

In conclusion, the lung cancer data presented here suggest strongly, although direct data on smoking behaviour are not available, that the smoking epidemic which started in England and Wales at the time of the First World War, has spread latest to women in rural parts of the northern counties of England, and that a particular focus for health education should be women in rural areas (and low socio-economic groups) in the north of the country.

### Methodological appendix

It is desirable in a case-control analysis using persons with disease as the controls that the comparison group should be formed by a wide variety of conditions so that any bias introduced by specific diseases should be minimised. If all cancer registrations except those for the site under study were used as the controls, a few cancer sites would dominate the controls at each age (e.g., lung for males and breast for females at many adult ages). An alternative approach which we have followed is to take a weighted sample of other cancers such that no single site contributes more than a small proportion of the total at each age.

In practice this was conducted as follows. For each sex, age and urbanisation stratum separately, the data for all counties combined were examined, and numbers of cancers in the commonest sites (at three digit level of the ICD), were iteratively reduced such that no single site contributed more than 7% of the total number of cases in that category. Because the process of reduction was based on a series of iterative calculations, the final percentage constituted by each of the commonest sites was not necessarily exactly 7% (but was always in the range 5–7%). For each cancer site, a stratum-specific weight was then obtained by dividing the number of cancers of the site in this amended (reduced) file by the corresponding number of cancers of the site in the original file. The set of site and stratum-specific weights was then applied to the original site-specific data in each county, to create a 'weighted' control file. The control group for case-control analysis of each individual cancer site consisted of all cancers in this 'weighted' control file except the one under study.

For example, for the stomach cancer analyses for urban males age 50–54, we started with 24,362 potential controls (Table I), amongst which two tumours (lung and non-melanoma skin cancers) represented almost 50% of the total. After weighting (Table I), no tumour contributed more than 5.8%; the numbers of controls contributed by less common sites remained unchanged, but their relative contribution was increased because of the reduction in the total number of controls.

**Table I** Stomach cancer control group for urban males aged 50–54 years: numbers and percentages of cancers from selected sites before and after weighting

| *Cancer site* | *Unweighted controls* *No.* | *(%)* | *Weighted controls* *No.* | *(%)* |
|---|---|---|---|---|
| Lung | 7,877 | (32.3) | 669 | (5.8) |
| Skin (non-melanoma) | 3,704 | (15.2) | 669 | (5.8) |
| Bladder | 1,539 | (6.3) | 669 | (5.8) |
| Oesophagus | 584 | (2.4) | 584 | (5.4) |
| Thyroid | 71 | (0.3) | 71 | (0.6) |
| Gum | 27 | (0.1) | 27 | (0.2) |
| All cancers (except stomach) | 24,362 | (100.0) | 11,594 | (100.0) |

We thank the Cancer Research Campaign who supported Dr Silva's work, the regional cancer registries who collected the cancer data and the Office of Population Censuses and Surveys who gave access to them, Mrs C. Little for data extraction, and Mrs P. Cook-Mozaffari for information on bracken distribution.

### References

BARKER, D.J.P., COGGON, D., OSMOND, C. & WICKHAM, C. (1990). Poor housing in childhood and high rates of stomach cancer in England and Wales. *Br. J. Cancer*, **61**, 575.

BLOT, W.J. & FRAUMENI, J.F. Jr (1982). Changing patterns of lung cancer in the United States. *Am. J. Epidemiol.*, **115**, 664.

BRESLOW, N.E. & DAY, N.E. (1980). *Statistical Methods in Cancer Research*. Vol. 1 – The Analysis of Case-Control Studies. IARC Scientific Publication No. 32. IARC: Lyon.

CAMPBELL, W.J. & NICHOLSON, A. (1989). *PC MAPICS*. Mapics Ltd: London.

DOLL, R. & PETO, R. (1981). *The Causes of Cancer*. Quantitative Estimates of Avoidable Risks of Cancer in the United States Today. Oxford University Press: Oxford.

GALPIN, O.P., WHITAKER, C.J., WHITAKER, Rh. & KASSAB, J.Y. (1990). Gastric cancer in Gwynedd. Possible links with bracken. *Br. J. Cancer*, **61**, 737.

GARDNER, M.J., WINTER, P.D., TAYLOR, C.P. & ACHESON, E.D. (1983). *Atlas of Cancer Mortality in England and Wales 1968–1978*. John Wiley: Chichester.

MANTEL, N. & HAENSZEL, W. (1959). Statistical aspects of the analysis of data from retrospective studies of disease. *J. Natl Cancer Inst.*, **22**, 719.

MINISTRY OF AGRICULTURE, FISHERIES AND FOOD (1957, 1963, 1967, 1974, 1980). Domestic/household food consumption and expenditure: 1955, 1961, 1965, 1972, 1978. *Annual Report of the National Food Survey Committe*. HMSO: London.

MINISTRY OF HEALTH (1936). Housing Act, 1935. *Report on the overcrowding survey in England and Wales 1936*. HMSO: London.

MINISTRY OF HEALTH (1939). *Report of the Committee of Inquiry into the Anti-Tuberculosis Service in Wales and Monmouthshire*. HMSO: London.

NOMURA, A. (1982). Stomach. In Schottenfeld, D. & Fraumeni, J.F. Jr. *Cancer Epidemiology and Prevention*, p. 624. W.B. Saunders: Philadelphia.

OFFICE OF POPULATION CENSUSES AND SURVEYS (1981). Area mortality. *The Registrar General's decennial supplement for England and Wales 1969–73*. Series DS No. 4. HMSO: London.

OFFICE OF POPULATION CENSUSES AND SURVEYS (1986, 1989). *General Household Survey 1984, 1986*. HMSO: London.

PHAROAH, P.O.D. & MORRIS, J.N. (1979). Postneonatal mortality. *Epidemiologic Reviews*, **1**, 170.

REGISTRAR GENERAL (1895). *Supplement to the Fifty-fifth Annual Report of the Registrar-General of Births, Deaths and Marriages in England*. Part I, HMSO: London.

REGISTRAR GENERAL (1903, 1912, 1920, 1922). *Sixty-fourth, Seventy-third, Eighty-first, Eighty-third Annual Reports of the Registrar-General of Births, Deaths, and Marriages in England and Wales* (1901, 1910, 1918, 1920). HMSO: London.

REGISTRAR GENERAL (1931, 1938). *The Registrar-General's Statistical Review of England and Wales for the Years 1930, 1937*. Tables. Part I, Medical. HMSO: London.

REGISTRAR GENERAL (1958). *The Registrar-General's Decennial Supplement, England and Wales, 1951. Area Mortality*. HMSO: London.

STOCKS. P. (1936). Distribution in England and Wales of cancer of various organs. *13th Annual Report of the British Empire Cancer Campaign*, p. 240.

STOCKS, P. (1937). Distribution in England and Wales of cancer of various organs. *14th Annual Report of the British Empire Cancer Campaign*, p. 198.

STOCKS, P. (1939). Distribution in England and Wales of cancer of various organs. *16th Annual Report of the British Empire Cancer Campaign*, p. 308.

STOCKS, P. (1947). Regional and local differences in cancer death rates. *Studies on Medical and Population Subjects*, No. 1. HMSO: London.

SWERDLOW, A.J. (1986). Cancer registration in England and Wales: some aspects relevant to interpretation of the data. *Journal of the Royal Statistical Society (A)*, **149**, 146.

SWERDLOW, A.J. & DOS SANTOS SILVA, I. (in preparation). *Cancer Research Campaign Atlas of Cancer Incidence in England and Wales, 1968–85*. Oxford University Press: Oxford.

WALD, N., KIRYLUK, S., DARBY, S., DOLL, R., PIKE, M. & PETO, R. (eds). (1988). *UK Smoking Statistics*. Oxford University Press: Oxford.

WORLD HEALTH ORGANIZATION (1967). *Manual of the International Statistical Classification of Diseases, Injuries, and Causes of Death*. Eighth Revision. WHO: Geneva.

WORLD HEALTH ORGANIZATION (1977). *Manual of the International Statistical Classification of Diseases, Injuries, and Causes of Death*. Ninth Revision. WHO: Geneva.

WORLD HEALTH ORGANIZATION (1979). Controlling the smoking epidemic. Report of the WHO Expert Committee on smoking control. *WHO Technical Report Series*, No. 636. WHO: Geneva.